# A novel mutation in *KDR* is associated with retinal venous beading and cerebral cavernous malformation

**DOI:** 10.1016/j.gendis.2024.101390

**Published:** 2024-08-13

**Authors:** Xiaonan Zhuang, Yuqiao Ju, Gezhi Xu, Xin Huang

**Affiliations:** a Department of Ophthalmology, Eye & ENT Hospital, Fudan University, Shanghai 200031, China; b Shanghai Key Laboratory of Visual Impairment and Restoration, Fudan University, Shanghai 200031, China; c NHC Key Laboratory of Myopia, Fudan University, Shanghai 200031, China

First described by Meredith in 1987, retinal venous beading (RVB) is an extremely rare and potentially vision-threatening disease,^1^ which is characterized by beading or sausage-like configuration of retinal veins. It can present with episodes of increased vascular permeability accompanied by lipid exudation, foveal edema, recurrent branch vein occlusion, retinal ischemia with retinal neovascularization, and vitreous hemorrhage,^1^ or exist solely and asymptomatically.^2^ Most reported RVB cases exhibited an autosomal dominant inheritance pattern, but some cases are still termed idiopathic or sporadic with no familial history.^2^ Up to now, the exact genetic background and pathogenesis of RVB are yet unclear.

Vascular endothelial growth factor receptor 2 (VEGFR2), encoded by the *KDR* gene, plays an important role in regulating angiogenesis and vascular development. Activation of VEGFR2 is known to mediate several key signaling pathways, including the phosphoinositide 3 kinase-protein kinase B (Akt)/Rac pathway and phospholipase C gamma (PLCγ)-protein kinase C-extracellular signal-regulated kinase (ERK)1/2 pathway. Dysregulation in VEGFR2 signaling has been implicated with retinal vascular defects^3^ and the formation of cerebral cavernous malformation (CCM).^4^ More recently, germline *KDR* variants have been found to be associated with tetralogy of Fallot.^5^

In this study, we report the clinical features and molecular characterization of a patient carrying a novel heterozygous missense variant of the *KDR* gene (c.1844C  >  T, p.T615I), providing evidence that the novel KDR variant contributes to the pathogenesis of vascular anomalies and is associated with RVB.

A 29-year-old man visited the Eye and ENT Hospital of Fudan University with a complaint of blurred vision in the left eye for about 1 year. The retinal vessels of his left eye showed alternately narrowing and dilation in all quadrants except the nasal inferior one, and were surrounded by large quantities of intraretinal and subretinal lipid exudates (Fig. 1Aa). The macula showed an orange-red hemangioma-like lesion (Fig. 1Aa, white arrow) and a round retinal cyst (Fig. 1Aa, white asterisk). The ultra-widefield fluorescein angiography examination revealed widespread “beading” with late staining mostly located along the branches of retinal veins (Fig. 1Ab, yellow arrows). The hemangioma-like lesion (Fig. 1Ab, white arrow) was observed in the terminal of one branch of a vein that exhibited slightly high hyperfluorescence surrounded by blocked fluorescence (bleeding). Multiple areas of telangiectasis (Fig. 1Ab, white arrowhead) were evident, and venous tortuosity (Fig. 1Ab, yellow arrowhead) was remarkable at the distal end of venous beading, which was suggestive of the increased venous return resistance. Therefore, we speculate that the structural defects of beading venous walls induced turbulence and stasis. The inherent structural weakness and additional endothelial injuries owing to increased venous pressure may eventually contribute to extravascular exudation, thrombosis, and even ischemia. Optical coherence tomography (OCT) B-scan confirmed a retinal cyst (Fig. 1Ad, white asterisk) at the level of the yellow line in the en-face OCT angiography slab of the left eye retina (Fig. 1Ac), and a hyperreflective lesion with blood flow signal was observed on the nasal border (Fig. 1Ad, e, white arrows). Along the line through the upper vessels on the confocal scanning super-brightness ophthalmoscope image (Fig. 1Af), the OCT B-scan showed that the calibers of beading retinal veins were larger and protruded into the vitreous (Fig. 1Ag, yellow arrow) as compared with those of concomitant arteries (Fig. 1Ag, green arrow). For the lower vessels, however, the proximal veins seemed normal and the difference between arteries and veins was not significant in the B-scan (Fig. 1Ah). In the right eye, the retinal arterioles were mildly tortuous but otherwise normal (Fig. 1Ai). The ultra-widefield fluorescein angiography examination revealed a mildly jagged appearance of the vascular walls of certain arteries and veins in the right eye (Fig. 1Aj, blue arrows).

**Figure 1:**
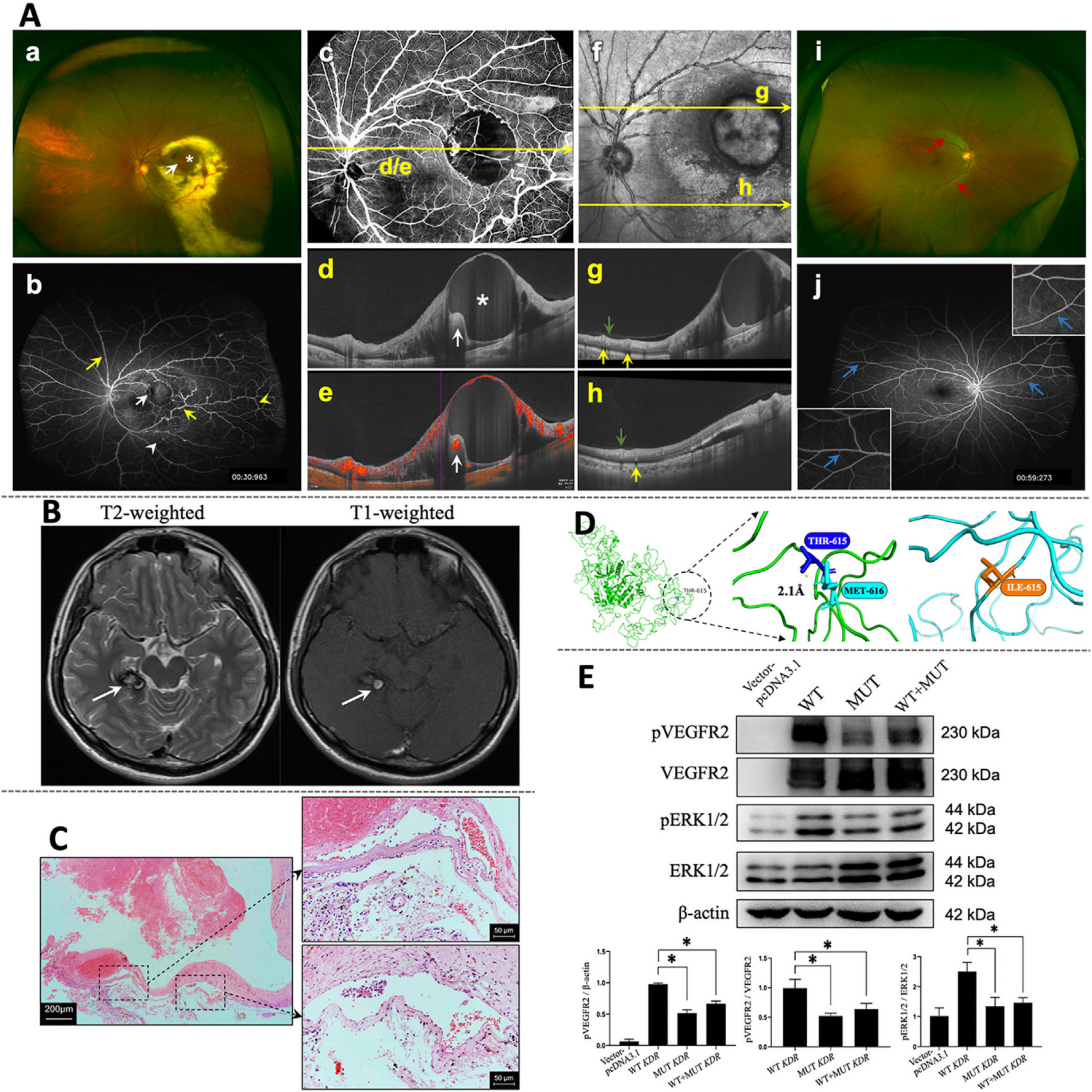
The germline mutation in *KDR* (c.1844C  >  T, p.T615I) is related to retinal venous beading and cerebral cavernous malformation. **(A)** Clinical evaluation of retinal vascular anomalies with multimodal imaging. (a, i) Ultra-wide photography shows extensive venous beading, exudates, a hemangioma-like lesion (a, white arrow), and a retinal cyst (a, white asterisk) in the left eye and arteriolar tortuosity (i, red arrows) in the right eye. (b, j) Ultra-wide field angiography shows late staining of the venous beading (b, yellow arrows), slightly high hyperfluorescence of the “hemangioma” (white arrow), venous tortuosity (b, yellow arrowhead), and telangiectasis (b, white arrowhead) in the left eye, and unsmooth appearance of certain vessels (j, blue arrows, boxes in the corners contain magnified views) in the right eye. (c) The en-face optical coherence tomography angiography slab of the retina in the left eye. (d, e) Taken along the level of the yellow line, the B-scan image shows a retinal cyst (d, white asterisk) and a hyperreflective lesion (d, white arrow) where the blood flow signal is evident in the angiography B-scan image (e). (f) The confocal scanning super-brightness ophthalmoscope image. Along the lines marked, the calibers of the beading veins (g, yellow arrows) are more dilated than those of the concomitant arteries (g, green arrow) in the upper part. For comparison, calibers of the veins (h, yellow arrow) and arteries (h, green arrow) in the lower part are present. **(B)** Magnetic resonance imaging (MRI) of the intracranial lesion. Brain MRI reveals a lesion located in the right temporal lobe, with heterogeneity of internal signal on T2-weighted and T1-weighted sequences. **(C)** Histological examination of the postoperative specimen. Hematoxylin and eosin staining and evaluation of postoperative sections reveal hemorrhage, fibrosis, and highly dilated cavernous vessels. Scale bar = 200 μm. The labeled areas of cavernomas are captured at higher magnification, respectively. Scale bar = 50 μm. **(D)** Three-dimensional structure of wild type and T615I-mutated VEGFR2 protein. The graph on the left presents the entire protein structure, and the interactions of Thr615 and its adjacent residue Met616 are highlighted. The hydrogen bond is represented by the dashed-yellow line, the length of which is shown along the dashed line in angstroms (Å). In the structure of wild-type VEGFR2 (green), the hydrogen bond is observed at a distance of 2.1 Å, while in the structure of T615I-mutated VEGFR2 (blue), Thr615 is substituted with Ile615.Thr, threonine; Met, methionine; Ile, Isoleucine. **(E)***KDR* mutation impairs VEGFR2 phosphorylation and VEGFR2-dependent signaling. Representative Western blot of HEK 293T cells transfected with an empty vector pcDNA3.1, wild-type *KDR* (WT), mutant T615I *KDR* alone (MUT), and mutant with wild-type *KDR* (WT + MUT). Quantitative analysis of pVEGFR2/β-actin, pVEGFR2/VEGFR2, and pERK1/2/ERK1/2 in different groups. *n* = 3; ∗*P* < 0.05.

The patient reported a history of neurosurgery owing to sustained headaches at the age of 17 years. The preoperative brain magnetic resonance imaging revealed a lesion in the right temporal lobe beside the brainstem. The lesion produced a heterogeneous internal signal on T2-weighted sequences and a T2 hypointense rim surrounding it (Fig. 1B, white arrow). The T1-weighted sequences demonstrated central hyperintensity mixed with isointensity (Fig. 1B). The lesion was mildly enhanced on contrasted T1-weight sequences (Fig. S1). The patient underwent an open surgery, and the pathological analysis of the resected specimen confirmed the diagnosis of CCM. In histological findings, the highly dilated sinusoidal vessels comprised a layer of endothelium and fibrosis but without smooth muscle cells (Fig. 1C). Hemorrhage and macrophages were also evident.

Aside from this event, the patient denied any other systemic disease, including diabetes mellitus and a family history of vascular disease. A careful full-body examination revealed no cutaneous lesion.

Genetic testing uncovered a novel heterozygous mutation (*KDR*: NM_002253, exon13, c.1844C  >  T, p.T615I). No other mutations highly suspected to be associated with vascular development or anomalies were found. Sanger sequencing results confirmed the mutation of the candidate gene *KDR* (Fig. S2). The affected residue is evolutionarily conserved across species. The mutation was predicted to be deleterious using multiple *in silico* prediction tools (Fig. S2). Bioinformatic analysis predicted the ability of this mutation to interfere with the formation of regional hydrogen bonds, which was shown in the three-dimensional structure of the wild-type and the mutated VEGFR2 protein (Fig. 1D).

*In vitro* studies were conducted to provide insight into the effects of the *KDR*: p.T615I mutation on VEGFR2 function and its downstream signaling pathways. The phosphorylation of VEGFR2 at the Tyr1175 residue mediates several key signaling pathways, including ERK1/2, which is vital for executing the biological effects of VEGFR2.^4^ Western blot analysis showed that the phosphorylated levels (Tyr1175) of T615I-mutated VEGFR2, with and without co-expression of wild-type VEGFR2 (WT VEGFR2), were dramatically lower than that of WT VEGFR2 (Fig. 1E). Further, the up-regulation in the phosphorylation of ERK1/2 induced by WT VEGFR2 was significantly remarkable than that induced by T615I-mutated VEGFR2, supporting the results regarding VEGFR2 phosphorylation (Fig. 1E). The phosphorylation level of Akt in cells transfected with T615I-mutated VEGFR2 was also significantly lower than that with WT VEGFR2 (Fig. S3). Noteworthy, the previous study demonstrated that several *KDR* variants found in tetralogy of Fallot could also reduce Tyr1175 phosphorylation.^5^ Therefore, we hypothesize that the mutation *KDR*: p.T615I can impair the execution of the biological function of VEGFR2 by resulting in insufficient Tyr1175 phosphorylation-dependent VEGFR2 signaling and contribute to structural defects of vascular walls, which can be manifested as venous beading or cavernous malformation.

Interestingly, among genes responsible for familial CCMs, namely *CCM1*, *CCM2*, and *CCM3*, CCM3 has been reported to interact with VEGFR2. We analyzed the previously reported *CCM3* truncating and missense mutations according to the ClinVar database. Most of the missense mutations lie in the functional domains of CCM3 protein, suggesting the pathogenicity (Fig. S4). Truncating mutations of *CCM3*, whose detailed information is listed in Table S1, make up the majority (81%) of reported *CCM3* mutations, with the underlying mechanism of loss of function (Fig. S5). Human truncating *CCM3* mutants destabilize VEGFR2 on the cell membrane, which consequently reduces VEGFR2 activity represented by Tyr1175 phosphorylation.^4^ The defects in VEGFR2/ERK signaling induced by human *CCM3* mutants may subsequently lead to disruption of endothelial cell junctions and vascular integrity, which contributes to the formation of cavernous malformation. Thus, *CCM3* mutations lead to vascular anomalies in the brain and retina, at least in part through inhibiting VEGFR2 signaling. Although the result of genetic testing in this patient was negative for pathogenic mutations of *CCM1*, *CCM2*, and *CCM3*, the crosstalk between VEGFR2 and CCM3 in the regulation of vascular development may account, at least in part if not completely, for the overlapping of the *KDR*-mutant phenotype with classic familial CCM.

In conclusion, we present herein a rare case of RVB and CCM harboring a novel variant of *KDR*. This is the first reported genetic evidence of RVB, which is long believed to have an autosomal dominant inheritance pattern. Our novel findings suggest that the *KDR* mutation may be associated with RVB and CCM, which expands the phenotype spectrum of *KDR* mutation. However, the current study is based on a single case, and further investigations are warranted to verify the phenotype and genotype correlations in *KDR*-associated diseases.

## Ethics declaration

This study was approved by the institutional review board (IRB) of the Eye and ENT Hospital of Fudan University, Shanghai, China, and conducted in accordance with the 1975 Declaration of Helsinki. The ethical approval number was [2020]2020119. An informed consent was obtained from the patient.

## CRediT authorship contribution statement

**Xiaonan Zhuang:** Conceptualization, Data curation, Formal analysis, Funding acquisition, Investigation, Methodology, Software, Validation, Visualization, Writing – original draft, Writing – review & editing. **Yuqiao Ju:** Conceptualization, Data curation, Formal analysis, Investigation, Methodology, Software, Validation, Visualization, Writing – original draft, Writing – review & editing. **Gezhi Xu:** Project administration, Resources, Supervision. **Xin Huang:** Funding acquisition, Project administration, Resources, Supervision.

## Conflict of interests

The authors reported no conflicting relationship.

## Funding

This work was supported by the 10.13039/501100001809National Natural Science Foundation of China (No. 82070975, 82301213), the Shanghai Science and Technology Commission of China (No. 21ZR1411400), and the Shanghai Hospital Development Center Foundation (SHDC12023116).
