# Supplementary material for: A novel mutation in KDR is associated with retinal venous beading and cerebral cavernous malformation
Source: Genes Dis. 2024 Aug 13;12(4):101390. doi: 10.1016/j.gendis.2024.101390 (PMC11981729; doi:10.1016/j.gendis.2024.101390)
Supplement: Multimedia component 1 [file mmc1.docx]

**Supplementary Material**

**Supplement Figure 1.** Magnetic resonance imaging of the brain on contrasted T1-weight sequences. The lesion was mildly enhanced on contrasted T1-weight sequences, but no associated developmental venous anomaly was identified.

**
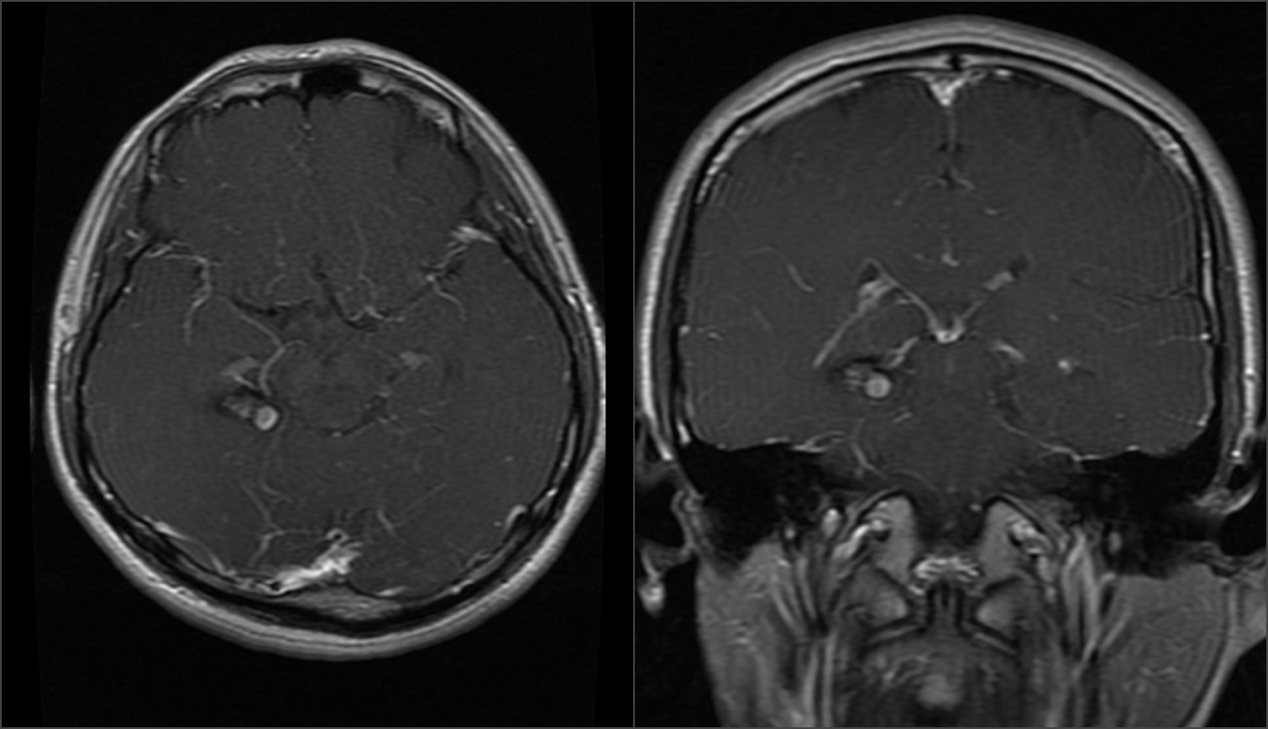
**

**Supplement Figure 2.** Identification and bioinformatic analysis of KDR mutation. (A) Sanger sequencing results confirmed the mutation of the candidate gene *KDR* (NM_002253, exon13, c.1844C>T, p.T615I). (B) Multiple sequence alignment of this mutation site (red arrow) from different species verified the conservatism. Amino acid similarity across species: Green100%; Blue≥75%; Pink≥50%; Red≥33%. * indicates high conservatism across species. (C) The mutation was predicted to be deleterious using multiple *in silico* prediction tools including Mutation Taster, SIFT (Sorting Intolerant From Tolerant), GERP (Genomic Evolutionary Rate Profiling), SPIDEX (A Pre-computed Index of Splicing Variants), ClinPred (Clinical prediction), LRT (Likelihood Ratio Test), Reference (the variant was not found in The 1000 Genome database, ExAC, and National Centre for Biotechnology Information, so it is considered novel), and ACMG (Grade of American College of Medical Genetics). According to SPIDEX analysis, the mutation was suspected to have no impact on mRNA splice during the transcription process.

**
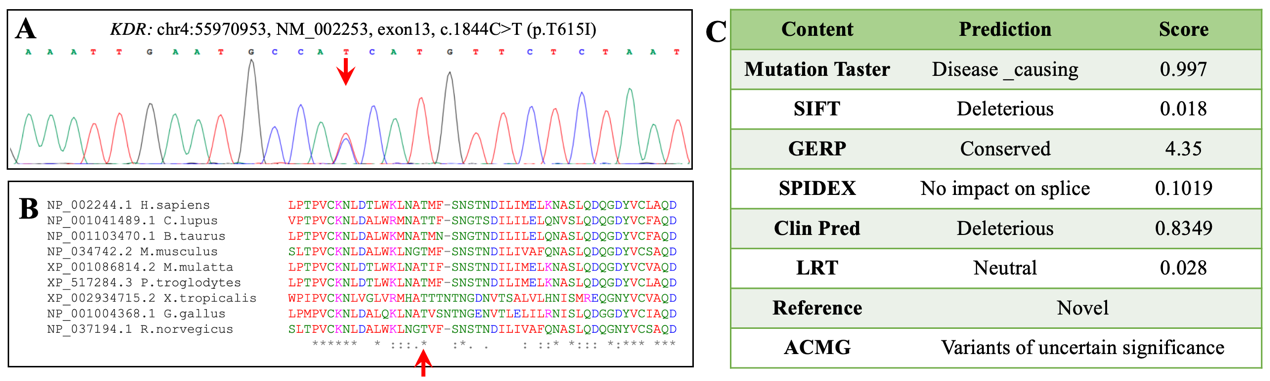
**

**Supplement Figure 3.** Effects of mutant T615I KDR on phosphorylation levels of Akt. (A) Representative western blot of HEK 293T cells transfected with wild-type KDR (WT) and mutant T615I KDR (MUT). (B) Quantitative analysis of pAkt/Akt in these groups. n = 3; **P* < 0.05.


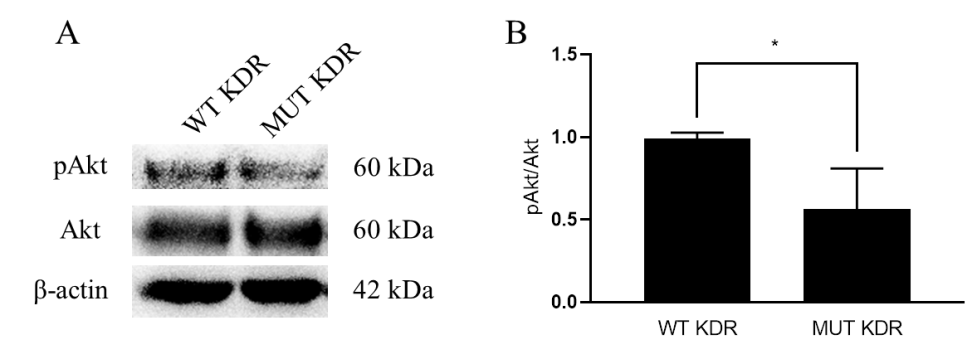


**Supplement Figure 4.** The distribution of CCM3 missense mutations (NM_007217.4) along the length of the CCM3 protein.

**
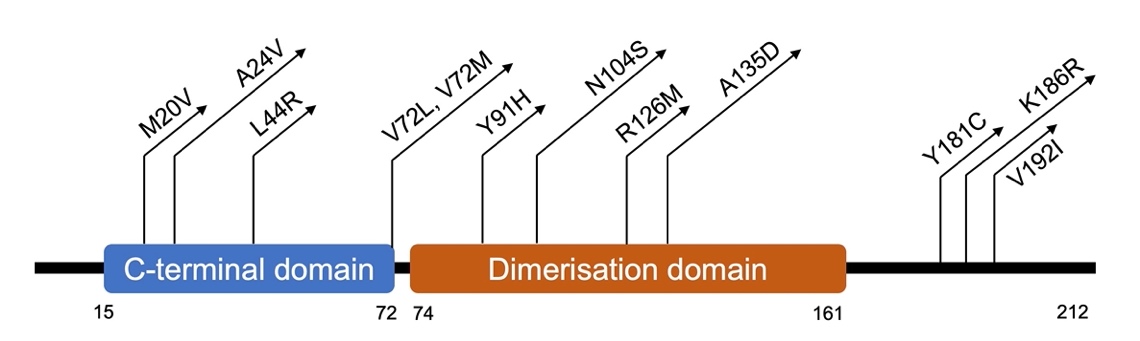
**

**Supplement Figure 5.** Overall distribution of *CCM3* genetic mutations (NM_007217.4) associated with cerebral cavernous malformation (CCM) in the ClinVar database according to mutation types.


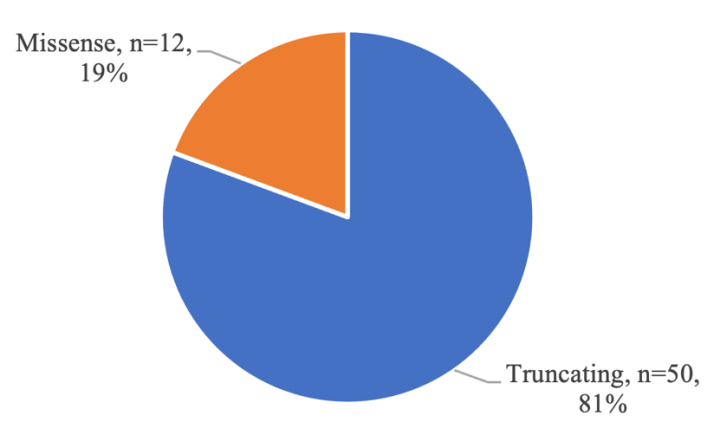


**Supplement Table 1.** Reported truncating mutations in *CCM3* gene (NM_007217.4) associated with cerebral cavernous malformation (CCM) in the ClinVar database.

| **Truncating type** | ***CCM3* gene mutation** | **Protein**  **change** | **Phenotypes** | **ACMG** | **Functional verification**^1^ |
| --- | --- | --- | --- | --- | --- |
| frameshift | c.62_71del | P21fs | CCM | P |  |
| frameshift | c.68_69del | Y23fs | CCM | P |  |
| frameshift | c.73_76dup | M26fs | CCM | LP |  |
| frameshift | c.131_132insTT | - | CCM | P |  |
| frameshift | c.160_161del | E54fs | CCM | P |  |
| frameshift | c.160_163del | E54fs | CCM | P |  |
| frameshift | c.164del | N55fs | CCM | P |  |
| frameshift | c.211del | S71fs | CCM | P |  |
| frameshift | c.211dup | S71fs | CCM | P |  |
| frameshift | c.229dup | T77fs | CCM | P |  |
| frameshift | c.243del | R82fs | CCM | P |  |
| frameshift | c.274_275del | M92fs | CCM | P |  |
| frameshift | c.333del | K111fs | CCM | P |  |
| frameshift | c.333dup | Q112fs | CCM | P |  |
| frameshift | c.334_337del | Q112fs | CCM | P |  |
| frameshift | c.351insT | R118fs | CCM | P | Verified |
| frameshift | c.392_395del | I131fs | CCM | P |  |
| frameshift | c.430dup | T144fs | CCM | P |  |
| frameshift | c.442_443del | V148fs | CCM | P |  |
| frameshift | c.522_528del | F174fs | CCM | P |  |
| frameshift | c.529dup | T177fs | CCM | P |  |
| frameshift | c.565_566del | N189fs | CCM | P |  |
| frameshift | c.575dup | S193fs | CCM | LP |  |
| frameshift | c.576_579del | S193fs | CCM | P |  |
| frameshift | c.584dup | N195fs | CCM | LP |  |
| frameshift | c.592dup | I198fs | CCM | P |  |
| nonsense | c.103C>T | R35* | CCM | P |  |
| nonsense | c.160G>T | E54* | CCM | P |  |
| nonsense | c.178C>T | Q60* | CCM | P |  |
| nonsense | c.208A>T | K70* | CCM | P |  |
| nonsense | c.283C>T | R95* | CCM | P | Verified |
| nonsense | c.301C>T | Q101* | CCM | P |  |
| nonsense | c.322C>T | R108* | CCM | P |  |
| nonsense | c.385C>T | Q129* | CCM | P |  |
| nonsense | c.394A>T | K132* | CCM | P |  |
| nonsense | c.418G>T | E140* | CCM | P |  |
| nonsense | c.456T>G | Y152* | CCM | P |  |
| nonsense | c.510C>G | Y170* | CCM | LP |  |
| nonsense | c.586C>T | R196* | CCM | P | Verified |
| splice | c.-117+6518_20del | - | CCM | P |  |
| splice | c.97_150del | - | CCM | P |  |
| splice | c.97-1G>A | - | CCM | P |  |
| splice | c.269-1G>C | - | CCM | LP |  |
| splice | c.396-2A>C | - | CCM | P |  |
| splice | c.475-1G>A | - | CCM | P |  |
| splice | c.557+1G>A | - | CCM | P |  |
| splice | c.557+1G>T | - | CCM | P |  |
| splice | c.557+4_557+7del | - | CCM | P |  |
| splice | c.558-2A>C | - | CCM | P |  |
| splice | c.558-2A>G | - | CCM | P |  |

**Supplement Methods and materials**

***Ocular examination***

The patient underwent standard ophthalmologic examinations, including best corrected visual acuity, slit-lamp biomicroscopy, intraocular pressure (IOP) measurement, B-ultrasound, ultra-wide field photography, and ultra-wide field fluorescein angiography (UWFA) (Optos 200Tx, Optos Plc., Dunfermline, UK), optical coherence tomography (OCT) (Spectralis, Heidelberg Engineering Inc., Heidelberg, Germany), and swept-source OCT angiography (SS-OCTA) (VG200D, SVision Imaging, Ltd., Luoyang, China).

***Genetic investigation and bioinformatic analysis***

Peripheral blood samples were obtained, and DNA was extracted from the whole blood sample using the FlexiGene DNA Kit (Qiagen, Hilden, Germany) as per the manufacturer’s instruction. The genomic DNA was subjected to whole-exome sequencing comprising more than 23000 genes (MyGenostics, Shanghai, China). On average, the mean coverage depth was 218.27, and the coverage of the target region was 98.08% (20X) and 98.92% (10X) in the sequencing system (Illumina, San Diego, USA). The candidate variants were further validated using Sanger sequencing as per the standard procedure. Polymerase chain reaction (PCR) was performed, and PCR products were sequenced using an ABI3730xl DNA Analyzer (Applied Biosystems, Foster City, USA). Amplified genomic sequences were compared with the *KDR* reference sequence NM_002253.

The databases National Centre for Biotechnology Information (https://www.ncbi.nlm.nih.gov), ExAC (http://exac.broadinstitute.org), and Exome Sequencing Project (https://evs.gs.washington.edu) were searched to determine if this variant had been previously reported. Further, the variant was subjected to bioinformatic and pathogenicity prediction analyses using diverse techniques as follows:

- Mutation Taster; http://www.mutationtaster.org,
- Sorting Intolerant from Tolerant, SIFT; http://sift.jcvi.org,
- Genomic Evolutionary Rate Profiling, GERP; http://mendel.stanford.edu/SidowLab/downloads/gerp/,
- A pre-computed Index of Splicing Variants, SPIDEX; http://www.openbioinformatics.org/annovar/spidex_download_form.php,
- ClinPred; https://sites.google.com/site/clinpred/,
- Likelihood Ratio Test, LRT; http://www.genetics.wustl.edu/jflab/lrt_query.html,

Conservativeness analysis was conducted using the European Molecular Biology Laboratory (EMBL)-European Bioinformatics Institute (EBI) (https://www.ebi.ac.uk/seqdb/confluence/display/JDSAT/Bioinformatics+Tools+FAQ). The variant was subsequently assessed according to the standards and guidelines of the American College of Medical Genetics and Genomics (ACMG). Three-dimensional structure modeling was carried out for human VEGFR2 encoded by wild-type *KDR* (NM_002253.2) and mutant *KDR* (p.T615I) using I-TASSER (https://zhanglab.ccmb.med.umich.edu/I-TASSER), and the constructed architecture was visualized using PyMOL (PyMOL Molecular Graphics System, Version 2.0, Schrödinger, LLC).

***Functional validation analysis of the novel mutation***

The human wild-type (WT) *KDR* and mutant (MUT) p.T615I-*KDR* plasmids were constructed in the pcDNA3.1 vector based on the reference sequence NM_002253 and the mutation information. The recombinant plasmids carrying the *KDR* fusion constructs were verified by direct DNA sequencing, amplified, and purified for transfection. The VEGFR2 protein was overexpressed in human embryonic kidney 293T cells, which show no endogenous VEGFR2 expression. 293T cells were cultured in Dulbecco’s modified Eagle’s medium (DMEM) supplemented with 10% fetal bovine serum (FBS) in a humidified incubator at 37°C under 5% CO_2_. Empty (pcDNA3.1), WT, MUT, or WT+MUT recombinant vectors were transiently transfected into 293T cells using the Liposomal Transfection 3000 Reagent (L3000015, Thermo Fisher Scientific, Waltham, USA) according to the manufacturer’s protocol. The cells were collected after 48 h of incubation and lysed in a cold lysis buffer (Beyotime, Shanghai, China) supplemented with phenylmethylsulfonyl fluoride (Beyotime) and protease inhibitor cocktail (Beyotime). The supernatants were collected from the centrifuged lysates, and the protein concentration was measured using a bicinchoninic acid (BCA) protein assay kit (Beyotime). Western blot (WB) analysis was conducted and the following primary antibodies were used, including rabbit anti-VEGFR2 (2479, Cell Signaling Technology, Danvers, USA), rabbit anti-phospho-VEGFR2 (Tyr1175) (2478, Cell Signaling Technology), rabbit anti-ERK1/2 (9102, Cell Signaling Technology), rabbit anti-phospho-ERK1/2 (Thr202/Tyr204) (9101, Cell Signaling Technology), rabbit anti-phospho-Akt (Thr308) (9275, Cell Signaling Technology), rabbit anti-Akt (4691, Cell Signaling Technology) and rabbit anti-β-actin (AF7018, Affinity Biosciences, Cincinnati, USA). WB results were analyzed by one-way analysis of variance (ANOVA), followed by Dunnett’s multiple comparisons test. A value of *P* < 0.05 was considered statistically significant.

***References***

1. He Y, Zhang H, Yu L, et al. Stabilization of VEGFR2 Signaling by Cerebral Cavernous Malformation 3 Is Critical for Vascular Development. Sci Signal 2010;3(116).
